# Correction: Premotor cortex hemodynamic responses primarily reflect perceptual rather than specific motor aspects of decision making

**DOI:** 10.1371/journal.pbio.3003849

**Published:** 2026-06-09

**Authors:** Jeff Boucher, Shihab Shamma, Yves Boubenec

S1–S8 Figs were uploaded incorrectly. Please see the correct S1–S8 Figs below.

## Supporting information

S1 Fig Behavior plots for both rules. A: Cartoon showing the organization of the AM pure tones in each rule for ferret B. “T” is a go stimulus, “R” is a reference stimulus. Ferret A would be the same, but with T and R locations reversed. B: Measured psychometric curves for ferret A, for each rule. C: Same as B, but for ferret B.
(EPS)

S2 Fig Demonstration of the method used for judging anatomical correspondences. In order to judge the anatomical location of any particular micromanipulator position, information from all anatomical images was taken into account. The images for each ferret were taken at anterio-posterior steps of 200 micrometers. Images on the left are anterior, and on the right are posterior. The AP position estimates can be used for comparison to pages 86, 92, 98, 104, and 110 of the Radtke-Schuller ferret atlas [17]. Particularly important landmarks in this case include the cruciate sulcus, which branches out from the interhemispheric fissure in a Y formation. Moving anterior from the branch point, it becomes more and more lateral. Also important and reliable as an anatomical anchor is the corpus callosum: when it is present, the large blood vessel in the intrahemispheric fissure has a “gap” in the image. Moving anterior, the corpus callosum is no longer present. This transition, according to the atlas, happens in between 26.1 and 25.5 mm anterior to the occipital crest. The example slice in the text was ferret A at −25.9. Green boxes surround the locations used for repeated recordings; an arrow at the bottom points to the recording location used as an example throughout the text.
(EPS)

S3 Fig Replication of example figures from Figs 1, 2, and 3 for all slices and both ferrets. The ferret and estimated slice AP position are noted on the left of each row. The example slice from the main text is in the second row. A, E, I, M: Percents correctly classified for each slice, as in Fig 1I. B, F, J, N: Projection plots as in Fig 2. Easy FA, Difficult FA, and Difficult CR projections are plotted on the same figure to save space. In addition, Difficult Hit and Easy Miss projections are included. C, G, K, O: Projection plots for Persistent/Restrained Lick trials among Difficult FA trials, as in Fig 3A. D, H, L, P: Projection plots for Persistent/Restrained Lick trials among Easy FA trials, as in Fig 3B.
(EPS)

S4 Fig Replication of example figures from Fig 4 for all slices and both ferrets. Same as S3 Fig, except after subtracting the regression model, as in Fig 4.
(EPS)

S5 Fig Consistency of anatomy and columns across recording sessions. Three example sessions are chosen for each ferret, using the same slice; the recordings were each taken approximately a week apart. Despite this, the gross anatomy looks the same for each image. Column positions were placed such that the positions of major vascular landmarks would be the same relative to each column; see, for example, the large vertical vessel under position “0” in ferret A, and the large vessel between columns 1.5 and 2.
(EPS)

S6 Fig Mean responses for all stimuli, regions, and ferrets. A, C, E, G, I: Mean Hit responses for each recording location for each ferret. Each of the six individual plots in a figure is a single stimulus; columns designate the distance from the boundary, while rows indicate the AM/freq rule. B, D, F, H, J: Same, but for CRs instead of Hits.
(EPS)

S7 Fig Persistent licks are reduced on Easy false alarm trials. A: Violin plots of the proportion of persistent licks in False Alarm trials across sessions for ferret A as a function of difficulty level. Each dot represents one session. Sessions with missing values or zero rates in either condition were excluded. B: Same as A for ferret B. C: Same as A for Hit trials (ferret A). D: Same as A for Hit trials (ferret B). A paired *t* test was performed within each panel to compare difficult versus easy trials, and significance is indicated above the violins (*p* < 0.05 * *p* < 0.01 ** *p* < 0.001 ***; n.s., not significant).
(EPS)

S8 Fig Design of the magnetic frame system for stabilizing the fUSI recordings against movements. A: Top view of the ferromagnetic frame. The frame was made of the corrosion-resistant, ferromagnetic stainless steel 440C. B: Bottom view of the ferromagnetic frame. The legs were used to aid in cementing by steadying the frame in place and providing locations to cement. C: Top view of the magnetic frame. The frame was made of aluminum, and designed to hold the micromanipulator and supermagnets while otherwise minimizing the space taken up. D: Bottom view of the magnetic frame. The supermagnets were stored in the three holes on the bottom; a later version (blueprint in Fig H) would have five holes for magnets. E: Photo of magnetic frame with micromanipulator and the probe-clamp. F: Photo of the 5-peg ferromagnetic frame. G, H: Detailed blueprints for the ferromagnetic frame (G) and magnetic frame (H) given to the metal shop which constructed them.
(EPS)

## References

[1] Boucher J, Shamma S, Boubenec Y. Premotor cortex hemodynamic responses primarily reflect perceptual rather than specific motor aspects of decision making. PLoS Biol. 2026;24(5):e3003768. doi: 10.1371/journal.pbio.3003768 42127153 PMC13170959

