# Supplementary material for: Premotor cortex hemodynamic responses primarily reflect perceptual rather than specific motor aspects of decision making
Source: PLoS Biol. 2026 May 13;24(5):e3003768. doi: 10.1371/journal.pbio.3003768 (PMC13170959; doi:10.1371/journal.pbio.3003768)
Supplement: Attachment — Submitted filename: Responses To Reviewers Boucher et al 2026.pdf [file pbio.3003768.s009.pdf]

Reviewer #1: This manuscript examines how stimuli are represented in the premotor cortex (PMC) of ferrets during decision-making, using functional ultrasound (fUS) imaging. The authors use a Go/NoGo delayed categorization task to disentangle whether premotor signals reflect stimulus perception or the eventual behavioral output. In the task, pure tone stimuli were categorized as 'Target' (requiring a lick response) or 'Reference' (requiring the animal to withhold licking). Task difficulty was manipulated by varying the distance of tones from the category boundary—tones farther from the boundary created easier trials, while those near the boundary created more difficult trials.

This gradation in difficulty was used to interpret error trials: errors on easy trials were assumed to be non-perceptual (i.e., due to lapses), while errors on difficult trials were interpreted as perceptual. fUS signals could robustly distinguish between easy Hit trials and Correct Rejections (CR), due to distinct cerebral blood volume (CBV) changes. These classifier axes were then used to project error trials. Easy errors were more similar to CRs, while difficult errors resembled Hits, suggesting that PMC activity reflects stimulus category representation rather than simply motor output. Licking behaviors, including "restrained" versus "persistent" licking, were carefully analyzed to support this interpretation.

Overall, this is an intriguing and well-motivated study. It leverages new neuroimaging technology in an animal model that is less commonly used, and the behavioral and neural analyses are thoughtfully structured. Below are comments to help strengthen the interpretation of the results.

We are glad to see that our core message has been so well understood. We will respond to your specific comments below.

Major comments

Behavioral Training Details.
The behavioral training description (e.g., Line 236) is unclear. Were the initial shaping sessions part of data collection? How long did it take to train the animals to criterion? What steps were involved? Also, there appears to be a notable number of false alarms even in the 'easy' stimulus region—was this expected? A 30-second timeout seems long; did this influence behavior or motivation?

We thank the reviewer for these questions. We now include a dedicated paragraph in the Methods detailing the training stages, their duration, and their relationship to the final dataset:

> *Initial shaping was intended to train animals on a different final task, so some specifics in the stimuli are different. Ferrets were trained on two pairs of stimuli, 2000 Hz and 16000 Hz tones modulated at 5.8 Hz for the frequency rule, and 5,657 Hz tones modulated at 2 Hz and 17 Hz for the AM rule. Rules were swapped automatically every 50 trials. Initially there was a 30 dB difference between Target (louder) and Reference stimuli. Ferrets took 1-2 weeks to demonstrate d' > 1 for this loudness-cued task, and would learn to*

perform the task more consistently and with no loudness cue in about 2 months. Ferrets were then trained on a task involving combinations of these stimuli for 2 months, after which the hypothesis for this paper was developed and the strategy changed. Ferrets were then trained to tolerate the 3 second delay; Ferret A took a month, Ferret B less than a week. Ferrets were then presented with the new stimuli. Ferrets instantly generalized when presented with single pairs per rule per session, but did not when there were multiple pairs per rule; due to this, the delay was reduced and ferrets were retrained to stable performance with d' > 1, in the soundproof booth where the final recordings would take place. Following this, they learned in a few sessions, and the delay was gradually reintroduced over the course of two weeks. All data collection took place following these steps.

To summarize, the initial shaping sessions were not part of data collection. They were performed to train the animals on an earlier version of the task (with different stimuli and a loudness cue) and to establish stable Go/NoGo behavior. Only sessions performed with the final stimuli and delay structure described in the main text contributed to the imaging dataset.

The FA rates in the present study are indeed higher than in some other work from our lab using the same booth (e.g. Chillale et al., 2023, where FAs were <15%). We note that this is typical in head-fixed Go/NoGo tasks with a motorized spout: the arrival of the spout itself reliably induces licking, and animals develop a strong bias toward the rewarded “Go” response. We deliberately exploited this bias to obtain a substantial number of putative non-perceptual FAs, which are central to our analysis. The 30 s timeout was introduced to counteract this tendency. Here, we chose to preserve the motorized-spout plus delay configuration because it is essential for capturing delay-related activity, accepting a higher FA rate as a trade-off. Importantly, our key comparisons are made within outcome classes (Hits vs FAs vs CRs), so the elevated overall FA rate does not compromise the main conclusions.

fUS Analysis Across Rule Types
Were the 'feature-rule' and 'amplitude-rule' blocks analyzed separately or pooled? If they were pooled, could this mask rule-specific effects? A separation might provide insight into whether PMC activity generalizes across task contexts.

In the original submission, data from frequency-rule and AM-rule blocks were pooled. We chose to pool them because we found no evidence that the current premotor dataset carried reliable information about the active rule. We now demonstrate this by plotting the average PMC responses to each stimulus separately for feature-rule and amplitude-rule blocks (now Supp Figure 6). These response profiles are highly similar across rules, with no systematic rule-dependent shift in CBV. Note this is consistent with representation of target stimuli irrespective of sensory modality found previously in ferret PMC (Fritz et al. 2010). These analyses suggest that pooling across feature-rule and amplitude-rule blocks is unlikely to mask strong rule-specific effects in PMC; if such effects exist, they are small relative to the categorical signals that are the focus of this study. The rationale was stated but not elaborated in the original submission; the relevant section now points to the Supp Figure as follows:

For every analysis, feature-rules are pooled together, as qualitatively the results within rules were indistinguishable (Supp Figure 6)

Voxel Averaging and Classifier Training

The approach of averaging voxels into columns for session pooling is an interesting strategy. However, it is unclear whether classifiers were trained per animal or on combined data. If classifiers were trained separately, does the column-averaging allow for cross-animal data integration? Clarifying this could help interpret the generalizability of the classifiers.

Thank you for pointing this out. All classifiers were trained per animal, and within each animal they were trained per region, defined by the anterior–posterior (AP) recording location (Supp Figure 2).

In principle, the column-averaging approach could support cross-animal data integration, but this would require accurate registration of AP recording locations across animals. In our dataset, the two ferrets only share a single common AP coordinate (−25.9 mm), so cross-animal pooling would be feasible for at most one pair of classifiers. We therefore did not attempt cross-animal classification, as it would provide only very limited additional insight and is not required for any of the conclusions we draw. Nevertheless, the PMC responses at this coordinate show qualitative similarity in space and time (Supp Figure 6). We replot below some of these responses suggesting that, with more data, cross-animal classification might perform above chance in a time-dependent manner. We have also added the following sentence to the Results section to clarify this:

Models were trained on each time bin and slice separately, allowing for tracking of decoding accuracy over the trial timecourse.

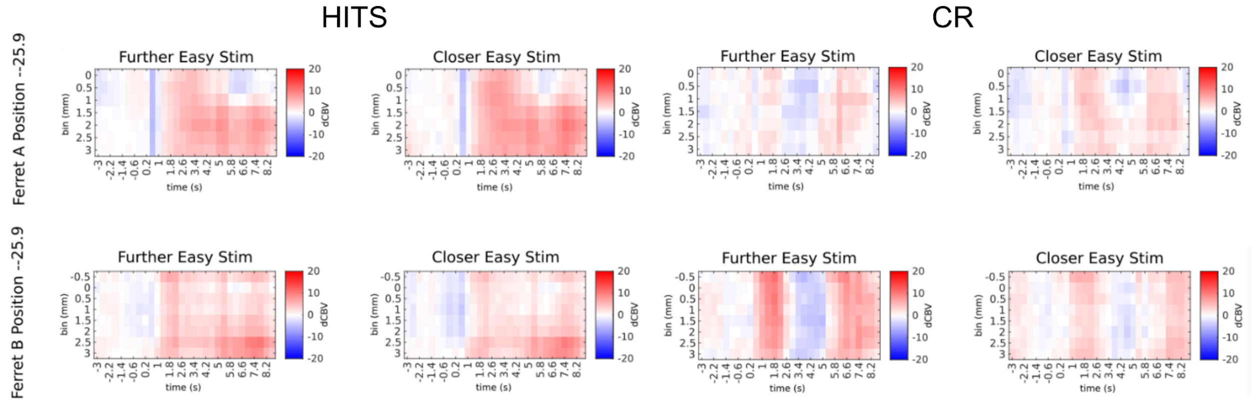

Comparison of hemodynamic response patterns across animals. Mean Hit and CR responses at the recording location shared across ferrets in the AM task.

PMC-Auditory Cortex Relationship

There is apparent stimulus-related classification accuracy shortly after tone onset (e.g., Figures 1I, 4C, and Supplementary Figure 6). Could the authors elaborate on potential auditory processing in PMC or rapid top-down feedback? Is there known auditory input to PMC in ferrets?

Regarding circuitry, premotor cortex is embedded in a broader fronto-parietal network that interacts with sensory cortices, and past works indicate that PMC sends feedback projections to auditory cortex (Fritz et al, 2010; Bimbard et al, 2018). These anatomical relationships make rapid top-down influences on auditory processing, and rapid access of task-related auditory information to PMC, plausible.

That said, we do not think that the early decoding peak reflects specific auditory feature coding in PMC. The response you are referring to is too fast to plausibly be locally neural, and is in addition a vasoconstriction rather than vasodilation (as can be seen in Fig. 1G).

Our preferred explanation for it is that it is a standard homeostatic reaction to arousal: the baroreflex. On arousal, blood vessels will reflexively tighten so that any sudden movements will not move the blood around and cause fainting (or “Orthostatic hypotension”). Classical conditioning can be used to cause animals to react to arbitrary stimuli by activating the arousal systems, and such effects should also be expected for operant conditioning like in this study (see Dworkin and Dworkin 1995 *Learning of physiological responses: II. Classical conditioning of the baroreflex*). So, perhaps there is a stimulus selective arousal that leads to a stimulus selective vessel-tightening reflex. We find this interesting, but somewhat tangential to the main focus of the paper, and so choose not to elaborate on this within the text.

Another possibility is that this transient vasoconstriction reflects a redistribution of blood flow early in the trial, wherein resources are prioritized for sensory regions at the time of stimulus processing and temporarily reduced in PMC. The latency of the effect is quite fast for a purely vascular reallocation mechanism, so we are cautious not to over-interpret it, and we cannot definitively distinguish this explanation from alternative accounts based on the current data. Such a situation would also exist simultaneously to the baroreflex.

CBV Decreases During Hits

What is the interpretation of the negative CBV deflections during Hit trials? Are similar spatial patterns observed during difficult trials (analogous to Figure 1E/G/H)? It would be helpful to directly compare CBV maps for Hits and CRs across difficulty levels.

We thank the reviewer for raising this point. We interpret the early negative CBV deflection during Hit trials as a transient vasoconstriction, plausibly reflecting a rapid redistribution of blood flow at stimulus onset (as discussed above).

Importantly, similar downward deflections are also present in difficult trials. This can already be seen in the population projections: difficult FA trials follow the Hit trials during the early post-stimulus period (Fig. 2C,D), indicating that this initial CBV decrease is not specific to easy trials.

Following the reviewer’s suggestion, we now directly compare CBV maps for Hits and CRs across difficulty levels and include these maps as Supp Figure 6. This comparison shows that the early negative deflection has a similar spatial pattern across easy and difficult conditions.

Licking Behavior Dynamics

Do restrained versus persistent licking behaviors vary across the session timecourse or depend on trial type (e.g., more persistent licking during Target trials or at later session stages)? This could influence interpretation of motor contributions to the signal.

We agree that potential changes in licking strategy over time or across trial types are important to consider when interpreting motor contributions to the neural signal. We therefore quantified the occurrence of persistent versus restrained licking both across sessions and within sessions, and as a function of trial type and difficulty.
First, across sessions, the overall rate of persistent licking did not show any obvious systematic trend: some sessions had slightly more persistent licks, some fewer, but there was no consistent increase or decrease over the course of training.

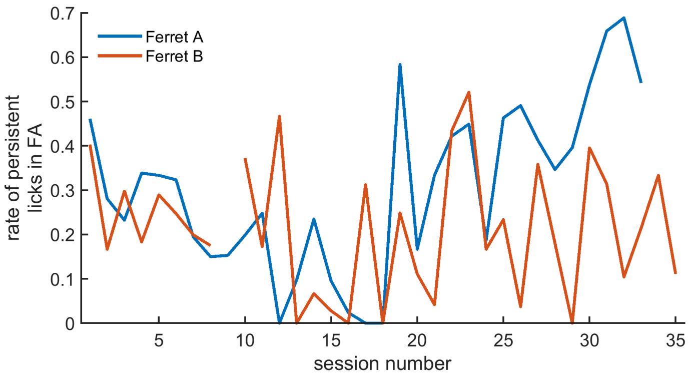

Second, within sessions, we examined the rate of false alarms (FA) over time and found that FA rates decreased as the session progressed (top panels). Consistent with this, the rates of FAs accompanied by persistent licking and by restrained licking both significantly decreased within session (bottom panels). Importantly, these two FA subtypes did not differ significantly from each other in their within-session timecourses. Thus, any within-session changes in motivation or motor vigor affect persistent and restrained licking in parallel, rather than selectively biasing one licking strategy.

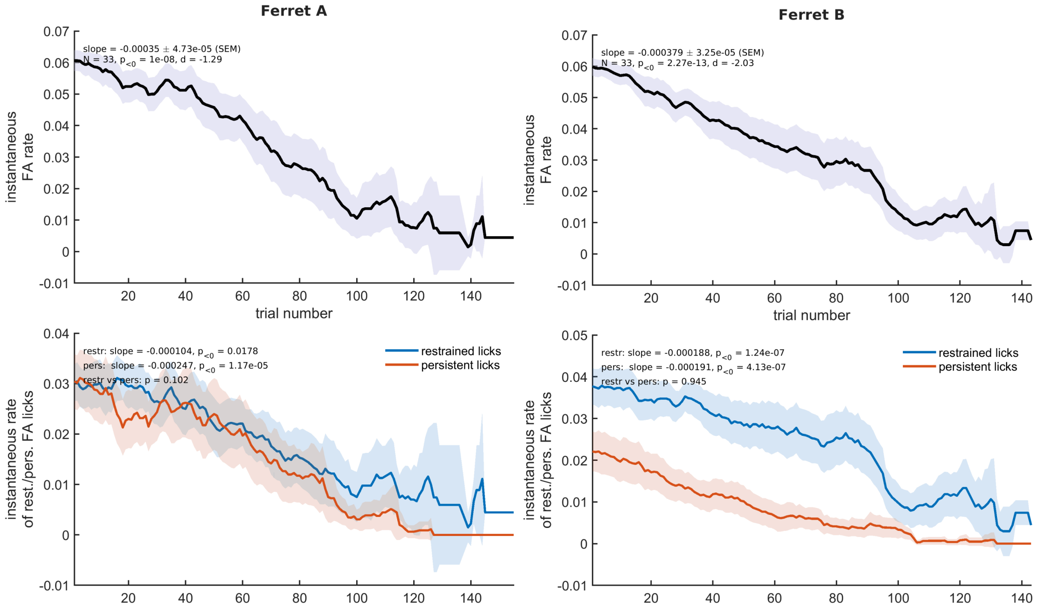

Third, we examined how persistent licking depends on trial type and difficulty. For false alarms (left panels), the rate of persistent licks was higher for difficult than for easy trials in both animals, as would be expected if persistent licking is more likely to reflect perceptual errors than restrained licking (as perceptual errors should be more likely in difficult trials). For hits (right panels), the rate of persistent licks was higher for easy than difficult trials in Ferret A, with the same trend but not significant in Ferret B, suggesting that stronger sensory evidence can also promote more vigorous licking on correct target detections.

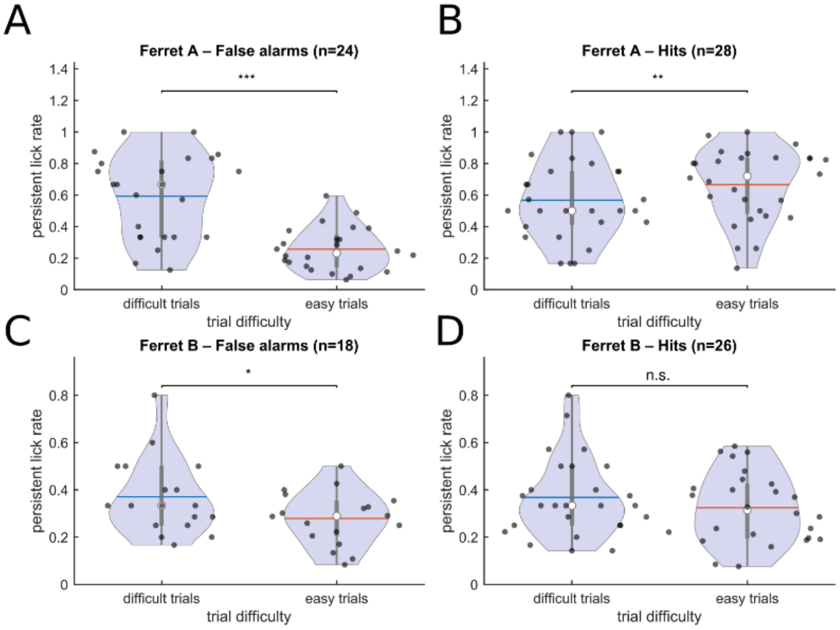

Taken together, these analyses show that (i) there is no systematic drift in persistent licking across sessions, (ii) within a session, persistent and restrained licking decrease in parallel over time, and (iii) trial-type effects on licking do not map straightforwardly onto the neural differences we report between premotor cortex and auditory cortex. While we cannot fully exclude some contribution of licking dynamics to the premotor signal, the absence of a selective temporal bias for persistent versus restrained licking makes a purely motor explanation of our findings unlikely. We now describe these results in the revised manuscript and added the above figure as Supp Figure 7:

> *To further validate the binarization into Persistent vs Restrained licking, we quantified how often trials were classified as Persistent as a function of trial type and difficulty (Supp Figure 7). For False Alarms, the fraction of Persistent-lick trials was higher for Difficult than for Easy stimuli in both animals, consistent with Persistent licking being more likely (though not exclusively) on trials where sensory evidence is weaker. For Hits, the proportion of Persistent trials was higher for Easy than Difficult stimuli in Ferret A, with the same but non-significant trend in Ferret B, suggesting that stronger sensory evidence can also promote more vigorous licking on correct detections. These distributions support the idea that our binary classification captures a behaviorally meaningful dimension of licking.*

Minor Comments
Define FA in Context
Line 45: Please define "FA trial" (false alarm) in the context of signal detection theory. This would help clarify the expected neural similarity to CR trials.

Thank you; we have edited the text as follows:

> *Meanwhile, False Alarm (FA) errors (i.e., incorrect "Go" responses to Reference stimuli) were also similar to CR trials*

Clarify 'Similar' in Line 54
Line 54: What aspect of Easy and Difficult CRs were 'similar'? CBV patterns? Behavioral outcomes? Please specify.

Done, thanks.

> *Hemodynamic response patterns for Easy and Difficult CR were similar*

Report Results for Difficult Targets
Line 69: For completeness, include the results from difficult Target trials, as these could inform the perceptual decision-making analysis.

Thanks for this; we have made the text much more complete:

> *Hit rates for Difficult Target stimuli were similar but slightly lower than those for Easy Target stimuli (ferret A: Hit rate 0.91, ferret B: Hit rate 0.90) suggesting that ferrets may not have actually found these stimuli difficult; no significant difference was found (ferret A p = 0.06, n = 183 Difficult Target trials, 1120 Easy Target trials; ferret B p = 0.06, n = 228 Difficult Target trials, 1353 Easy Target trials, one-tailed two sample z-test). An inability to find a significant effect here may have had to do with the ferrets' low decision criterion, leading to a lack of difficult miss trials to analyze (Ferret 1 n = 17 Difficult Misses, Ferret 2 n = 22 Difficult Misses). For these reasons, analysis of difficulty effects will focus on Reference stimuli and False Alarm trials.*

Clarify Figure 1 Legend
The legend for Figure 1 is unclear. Does "example pair of two reference stimuli" refer to the two 'easy' reference stimuli from the two-frequency rule?

Thanks for your feedback. Yes, the caption does refer to what you think. We have added additional language to the caption to clarify this. Additionally, the new Supp Figure 6 allows one to evaluate this claim for any pair of stimuli as they wish:

> ***J:** same as I, but comparing the two easy reference stimuli for the frequency rule. This example pair was chosen arbitrarily; all other pairs show similarly negative results, supporting the interpretation that responses are categorical.*

Typo Correction
Line 292: "Vowel averaging" should be "voxel averaging." Please correct this typographical error.

Done, thanks.

Reviewer #2: This study investigates the role of the premotor cortex (PMC) in auditory categorization using functional ultrasound imaging in ferrets performing a Go/No-Go delayed categorization task. It provides novel and valuable insights into the types of signals encoded in the PMC—choice versus perception—in response to task stimuli. This information is relevant to perceptual decision-making research, as it challenges assumptions that all behavioral task errors are purely perceptual, or that errors in perceptual tasks are idiosyncratic and uninterpretable. The clear differentiation of error types represents a novel contribution. While the experimental design and methodology are generally sound, additional feedback from researchers familiar with the functional ultrasound imaging technique could strengthen the manuscript.
The design of the behavioral task, combined with neural activity recordings, effectively demonstrates that errors occurring with stimuli closer to the category boundary (Difficult-Ref) are differentially encoded in the PMC compared to errors occurring with stimuli further from the boundary (Easy-Ref). Specifically, Difficult-Ref FAs are more similar to Hit trials, while Easy-Ref FAs resemble Correct Rejections.
However, some aspects of task design remain unclear, potentially leading to alternative interpretations of the main conclusion regarding representation of "subjective stimulus category." Incomplete data presentation further complicates full evaluation and interpretation of the claims. Enhanced descriptions of experiments and clearer result presentation would better support the conclusions.

Thank you for your feedback. We are glad you appreciate and have understood the core message. We will attempt to address your concerns with our data presentation and clarity as we address your specific comments below.

Major:
1. Task design and reward expectation: Why is reward provided only for Hit and not CR trials, creating an asymmetric reward schedule and reward expectation scenario? Only licking and targets yield rewards, making perceptual category representation potentially confounded with reward expectation. Could the authors clarify the reasoning behind this task design? How can reward expectation and sensory processing be distinguished? Why is the task described as detection rather than discrimination?

We thank the reviewer for raising these important points. We used a Go/NoGo design with reward delivered only on Hits because this is a standard appetitive auditory task in non-human animals, which typically involves an asymmetric reward schedule between the two stimulus categories (e.g., Fritz et al., 2003; Yin et al., 2020). We were fully aware of the limitations associated with such asymmetry, but chose this design precisely because it tends to bias animals toward making non-perceptual false alarms (i.e. “impulsive” Go responses), which are central to our question about disentangling internal category from reported choice. A substantial literature shows that animals in decision tasks exhibit action biases toward the option associated with reward (Brunton et al., 2013; Busse et al., 2011; Moore et al., 2023), and this is especially pronounced in appetitive Go/NoGo paradigms.

We agree that, in this setting, reward expectation and sensory categorization cannot be cleanly disentangled. At the same time, prior work suggests that both reward expectation and categorical sensory decisions are represented in frontal areas (Fritz et al, 2010); so it could be that both contribute to the premotor signal we observe. Importantly, however, our key result is that premotor activity tracks the animal's *internal category* (i.e. whether it treats the stimulus as "target-like") rather than the executed lick. Reward expectation in this task is itself largely determined by that internal categorical belief (a stimulus internally categorized as "target" predicts possible reward if the animal licks), so the two constructs are tightly linked rather than purely competing explanations. We now more explicitly acknowledge this limitation and clarify that our data do not allow us to fully separate reward-related from categorical components of the premotor signal, and that a future study using a symmetric two-alternative design (possibly combined with a NoGo condition) would be required to do so:

> *Thus, our findings reflect integrated neural population responses rather than individual neuron activity. In line with previous work showing that neuronal response properties in ferret frontal cortex are varied and not strictly anatomically localized (Fritz et al 2010), it is likely that the region of PMC we studied contains mixed-selectivity neurons jointly sensitive to sensory category, action and reward expectation, as previously reported in rodents and primates (Reinert et al 2021, Fan et al 2017). Our data demonstrate that at the hemodynamic level PMC activity tracks the animal's internal auditory category, but they cannot specify the relative contribution of these different neuronal subpopulations.*
> *A natural next step is therefore to use targeted electrophysiology guided by these fUSI maps. Based on the present results and existing work in frontal cortex, we would expect single-unit and population recordings in ferret PMC to reveal neurons whose firing (i) tracks the internal auditory category even when report and category diverge (e.g. Easy FAs), and (ii) more closely reflects reward anticipation or motor preparation. Our fUSI data identify specific PMC columns and time windows where such signals are likely to be strongest. Additionally, such a study could make use of a two-alternative forced choice paradigm, in which both sensory categories are rewarded to better dissociate sensory category signals from pure reward expectation, albeit potentially at the cost of reducing the number of non-perceptual errors that our current Go/NoGo design exploits.*

Finally, we agree that the task is more accurately described as a *discrimination* between two stimulus categories than as a detection task. We have revised the manuscript to consistently describe the paradigm as a discrimination task and to clarify the rationale and implications of the asymmetric reward schedule.

2. Interpretation of FAs: The authors claim Easy-Ref false alarms mainly reflect non-perceptual errors, whereas Difficult-Ref false alarms reflect perceptual errors. This claim appears overstated, considering that the FA rate for Easy-Ref trials is high (~50% for A, ~60% for B; Fig. 1C), and Difficult-Ref false alarms are about ~10 points higher. Generative models would thus predict that most Difficult-Ref FAs remain non-perceptual. Please clarify the reasoning behind these statements and provide estimates of perceptual FAs for each category. Is additional stratification of Difficult-Ref FAs possible?

We thank the reviewer for this careful point. Our intention was not to claim that Easy-Ref FAs are purely non-perceptual and Difficult-Ref FAs purely perceptual, but that perceptual errors are more likely on difficult trials. We recognize that our wording sometimes suggested a categorical interpretation. We have added additional sentences to clarify this at the end of the relevant results section, as follows:

> *Note that, across difficulty levels, the total error rate reflects a mixture of perceptual and non-perceptual errors, with the proportion of perceptual errors increasing with difficulty but never reaching 100%. Even so, this shift in the underlying mixture is detectable in the mean PMC response, leading to different average activity patterns for Easy versus Difficult False Alarms (Figure 1A).*

We have also adjusted figure 1 (adding figure 1A) to illustrate this more explicitly than the former figure 1B.

Conceptually, we fully agree with the intuition: Difficult-Ref FAs should be viewed as the sum of a similar pool of non-perceptual errors plus an additional contribution from perceptual errors. This is precisely why we focus on shifts in the mean projection rather than attempting to label individual FA trials as perceptual vs non-perceptual. Our data do not permit a decomposition of FA rates into these two components without strong model assumptions, so we now refrain from quoting specific fractions, instead framing our conclusions in relative terms.

Regarding additional stratification, we agree this is a promising direction and consider Fig. 3 as a first step. Based on behavioral observation, we found that Difficult-Ref FAs are more often accompanied by persistent licking, whereas Easy-Ref FAs more often involve a single lick followed by self-restraint. Figure 3C shows that when one stratifies by this licking style, one finds that the mean projection is closer to hit on persistent lick trials than restrained lick trials, which is consistent with the idea that licking style can titrate out perceptual errors more effectively. However, we emphasize that this too is a probabilistic marker, as persistent licking can also arise from frustration or disengagement (perhaps explaining the majority of persistent licks in easy FA trials, since those trials do not project similarly to hit trials). We have added additional text in the Discussion to clarify this:

> *Our current data cannot cleanly decompose these contributions, but they suggest that PMC provides a relatively stable internal category signal, while non-perceptual errors reflect how this signal is gated by state-dependent circuits in other frontal and parietal areas. Future work combining licking kinematics with independent markers of internal state (e.g. pupil size, heart rate) and targeted recordings in orbitofrontal and parietal cortices will be needed to disentangle action bias, exploration and attentional lapses as distinct sources of non-perceptual errors.*

3. Psychometric curve: Descriptions of Difficult-Ref and Easy-Ref trials use terms such as "slope" and "saturated," which are not clearly defined. Provide a quantitative description and clear definition of slope, saturation, and trial types (Ref, Target, difficulty levels) within the psychometric function.

We agree that these terms needed clearer definitions and have now clarified them in the Introduction and Results as follows:

> *[...] an error trial with a stimulus on the slope (central part) of the psychometric curve (a Difficult stimulus) should be relatively more likely to involve a perceptual error (Figure 1A); in this case, the stimulus would be internally classified as the incorrect category. For stimuli in the saturated regions (flat parts) of the psychometric curve (Easy stimuli) [...]*
>
> *[...] Performance was saturated (flat/unchanging) [...]*

Qualitatively, we use “slope” to refer to the central, non-flat part of the psychometric curve where performance changes with stimulus value, and “saturated” to refer to the flat regions where performance has reached a plateau. Quantitatively, in the revised Results, we now state explicitly that a pair of adjacent stimuli was considered to lie on the slope if performance differed significantly between them and the easier stimuli, and in the saturated region if there was no significant change in performance between the two extreme stimuli:

> *FA rates for Reference stimuli were significantly higher for Difficult stimuli compared to Easy stimuli (Figure 1C), suggesting they were in the sloped part of the psychometric curve (ferret A $p < 10^{-5}$, $n = 193$ Difficult Reference trials, 1182 Easy Reference trials; ferret B $p = 0.004$, $n = 235$ Difficult Reference trials, 1409 Easy Reference trials, one-tailed two sample z-test).*

We also now explicitly define Ref and Target stimuli and how Easy-Ref and Difficult-Ref are chosen relative to this psychometric function (Easy-Ref in the saturated region, Difficult-Ref on the slope), so that the link between difficulty and the underlying psychometric curve is unambiguous:

> *[...] in which the ferrets needed to lick/Go after "Target" stimuli and refrain from licking on "Reference" trials*

4. Baseline differences between groups: In Fig. 1H, the data appear similar to Fig. 1G but shifted back by 1-2 seconds. Could the authors clarify whether this is intentional or an error, and explain the presence of inhibition specifically before sound presentation in Correct Rejection trials?

We thank the reviewer for raising this point. The apparent similarity between Fig. 1G and 1H is neither intentional nor an error. The two panels depict different trial groups, plotted with the same time alignment and the same color scale, but with clearly different absolute values; thus,

they cannot be simple time-shifted versions of one another, even if the overall shape of the time courses looks similar.
Regarding the apparent “inhibition” before sound onset in Correct Rejection trials, this is a visualization artifact of the colormap. We have verified that there is no consistent pre-stimulus CBV decrease in CR trials. Instead, the pre-stimulus values in this period are very close to zero and fluctuate around baseline; the slight blue tint reflects small, non-systematic deviations that are rendered as negative by the color scale.

Minor:
5. Behavioral task: Please provide clearer explanations of the two task versions (AM vs. FM rule) and clarify the "block logic." Although the methods state similar results across task versions, psychometric plots for each task type and animal would ne important (as is standard in the field). Clearly define parameters determining Easy-Ref and Difficult-Ref trials.

Thank you for these helpful suggestions. We have revised the Behavioral Task section (Methods 5.2) to more clearly describe the two task versions (AM vs. frequency rule), the corresponding stimulus sets, and how blocks were structured. We now explicitly state that the block logic is a vestige of an earlier training paradigm and clarify how blocks were used in the final task.

We have added per-rule, per-animal psychometric curves in what is now Supp Figure 1, as requested. In addition, we have included a cartoon illustrating which stimuli were designated difficult and which stimuli were designated easy. The absolute parameters of these stimuli were originally chosen based on past data, and has been clarified in the methods section as follows:

> *The choice of the distance between the difficult stimuli and the boundary was decided using the past behavioral results in Yin et al 2020, as the paper included data on behavioral performance for both AM rate discrimination and frequency discrimination.*

This categorization is crucially validated by the significantly different performance on the difficult Ref stimuli, as reported in the text.

6. Include more details of task stimuli and organization within the main text (currently only available in supplementary data), specifying clearly what AM and FM refer to, block organization, and definitions of Easy-Ref and Difficult-Ref stimuli (dB and Hz). Clarify differences between ferrets 1 and 2 in terms of target/reference stimuli in a cohesive section.

Thank you for these suggestions. To keep the Results focused on the main conceptual findings, we have chosen to place the detailed numerical parameters and task organization in the Methods; we had not, however, made this explicit in the Results, and so have added the following text:

> *Specific properties of these stimuli are elaborated in the methods section and Supp Figure 1A, but are not critical to our presentation of the conclusions.*

Supp Figure 1A was also added to aid the clarity of the explanation.
Finally, within the Methods section, we had neglected to mention the dB value of our stimuli, which was a constant 65 dB. We now state this.

7. Regarding licking, clarify the validity of binarizing each trial into "persistent" vs. "restrained" (piezoelectric sensor activation >30 ms during 200-400 ms after the response window). E.g., provide histograms or similar visualizations to validate the approach. Is it indeed the case (as needed for the argument), that persistent licking between Easy-Ref and Difficult-Ref FAs is similar (e.g., lick counts, bouts)?

Thank you for this helpful suggestion. Figures 3A and 3B already illustrate how “persistent” versus “restrained” licks appear in individual trials for Easy-Ref and Difficult-Ref FAs, and we chose rasters to allow the reader to appreciate trial-to-trial variability. These examples show that while persistent licks are relatively more frequent in the difficult condition, their qualitative structure (lick bouts and timing) is similar across Easy- and Difficult-Ref FAs. Following your comment, we performed a new supplementary analysis in which we quantify licking more directly. We now plot the distribution of lick bouts for Easy and Difficult FAs and find no significant difference between them in either animal, confirming that persistent licks in Easy and Difficult FAs are comparable.

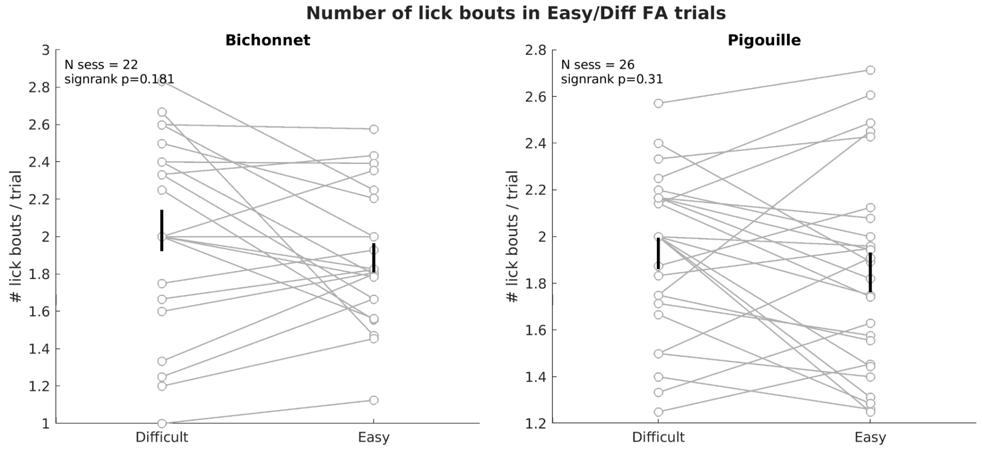

In addition, to validate further the binarization into persistent and restrained licks, we tested how the proportion of trials classified as persistent depends on trial type and difficulty. For false alarms, the fraction of persistent licking trials is higher for difficult than for easy stimuli in both animals, consistent with the idea that persistent licking is more likely (though not exclusively) associated with perceptual errors when sensory evidence is weaker. For hits, the proportion of persistent licking is higher for easy than difficult trials in Ferret A, with the same but non-significant trend in Ferret B, suggesting that stronger sensory evidence can also promote more vigorous licking on correct detections. We now describe these analyses in the Results and present the corresponding distributions and summary statistics in the new Supp Figure 7:

> *To further validate the binarization into Persistent vs Restrained licking, we quantified how often trials were classified as Persistent as a function of trial type and difficulty (Supp Figure 7). For False Alarms, the fraction of Persistent-lick trials was higher for*

Difficult than for Easy stimuli in both animals, consistent with Persistent licking being more likely (though not exclusively) on trials where sensory evidence is weaker. For Hits, the proportion of Persistent trials was higher for Easy than Difficult stimuli in Ferret A, with the same but non-significant trend in Ferret B, suggesting that stronger sensory evidence can also promote more vigorous licking on correct detections. These distributions support the idea that our binary classification captures a behaviorally meaningful dimension of licking.

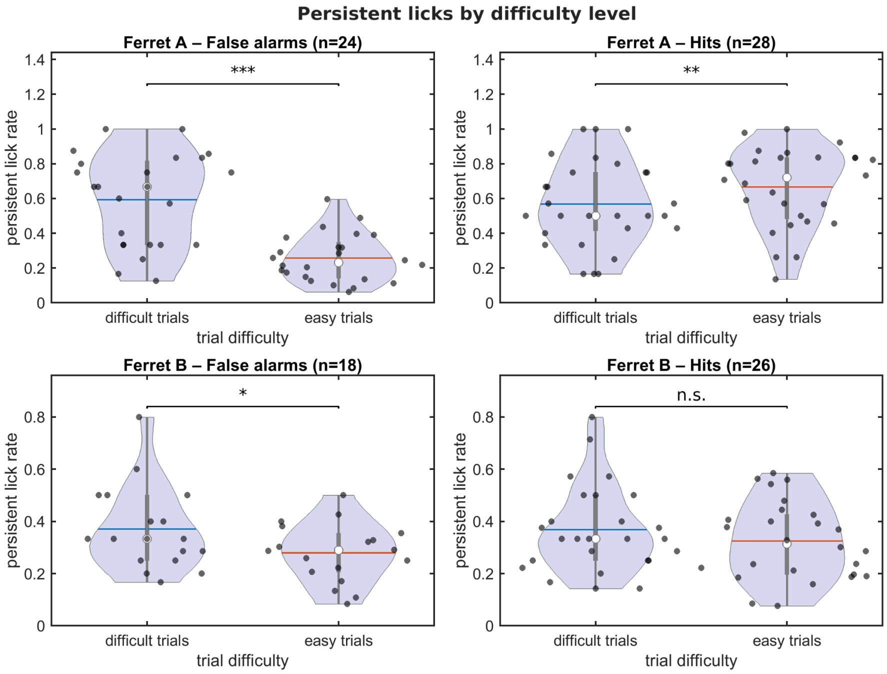

8. Provide additional explanation of PMC columns recorded are important: Could the authors clarify whether there are activity differences within PMC by depth, especially since Supplementary figures for ferret 2 differ from main text figures for ferret 1. Please justify presenting data from only one animal and single depth in the main text.

Thank you for this comment. We believe there may be a misunderstanding of our terminology. The different "slices" shown in the figures do not correspond to different depths within PMC but to different anterior–posterior (AP) positions. Each slice contains the full cortical depth in that coronal plane; what changes from slice to slice is AP location (slices are ~800 μm apart, all within anatomically defined PMC). We have now clarified this point explicitly in the Methods and adjusted Supplementary Fig. 2 to make it clear that we are varying AP position, not depth. Given that these are distinct AP locations, it is expected that the precise spatial activation patterns differ between slices, and we view it as informative that the main categorical effect and its time course are nevertheless robust across positions within a given ferret. A new Supp Figure 6, added in response to another comment, further illustrates this similarity across slices.

Our rationale for focusing on one animal and one AP position in the main panels is already stated in the main text: "For clarity, examples throughout this paper focus on one PMC position

from ferret A (17 sessions); results generalized across positions and animals (see supplementary figures).” We keep the main figures as simple as possible to highlight the core effects, while the supplementary figures show the corresponding maps and time courses for other AP positions and for ferret B. In addition, the main-text violin plots pool across all PMC slices and are plotted separately for each animal, demonstrating that the key effects are significant in both ferrets. We feel that adding multiple line plots for all slices and both animals to the main figures would substantially reduce clarity without changing any conclusions.

Regarding the differences between ferret 1 and ferret 2, these are primarily differences in effect magnitude, not in the presence or absence of the effect. As can be seen in Figures 2E and 3E, ferret 2 shows significant categorical signals for difficult FAs and even stronger effects when stratifying by licking behavior. Some behavioral differences between animals (e.g. how “difficult” the difficult stimuli are in practice) likely contribute to these magnitude differences, but we do not consider a detailed single-animal behavioral dissection to be appropriate to the current paper. The important point is that all key qualitative findings reported for ferret 1 generalize across PMC positions and are also present, and statistically significant, in ferret 2.

9. No data shown for miss trials: How does PMC activity during Miss trials appear, especially within the Hit/CR encoding space used in Fig. 2? Plot Miss trials for comparison with Hit, CR, and FA trials.

Thank you for your feedback. We have now included the Easy Miss projections for all slices and ferrets in Supp Figures 3 and 4. We describe at the end of the Results (and shown in Fig. 4H), lick-corrected Easy Miss trials project significantly closer to Hit than to CR relative to Easy FA trials, as expected if PMC primarily reflects the reward-predictive perceptual category (Target) even when the animal fails to lick. We now highlight this in the text:

> *In addition, performing the regression subtraction revealed that Easy Miss trials project more closely to Hit trials than to CR trials relative to Easy FA trials, as would be predicted if the activity reflected the reward-predictive categorization (Ferret A $p = 0.01$, Ferret B $p = 0.001$) (Figure 4H). Without accounting for licks, the effect was insignificant (Ferret A $p = 0.07$, Ferret B $p = 0.07$), potentially as a result of the large difference in mean lick rate between Hit and Miss trials (Fig 4B).*

This text comes in addition to an adjustment of Figure 4B and the addition of of a new panel in Figure 4 (FIgure 4H):

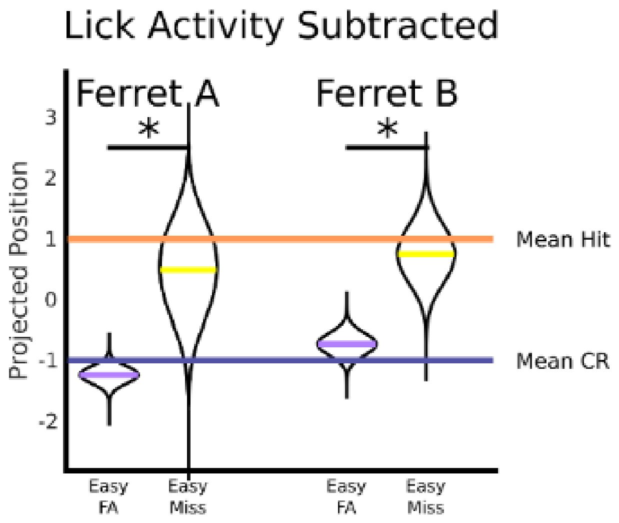

We have not included the Difficult Misses because there are far too few of them for anything reasonable to be concluded (Ferret A n = 17 Difficult Misses, Ferret B n = 22 Difficult Misses, both counts are aggregated across all slices and sessions). We have reported these counts in the text as follows:

> *An inability to find a significant effect here may have had to do with the ferrets' low decision criterion, leading to a lack of difficult miss trials to analyze (Ferret 1 n = 17 Difficult Misses, Ferret 2 n = 22 Difficult Misses). For these reasons, analysis of difficulty effects will focus on Reference stimuli and False Alarm trials.*

10. Is there evidence supporting the idea that additional waiting during reference stimuli represents a "punishment" (as stated) for ferrets?

We agree that our wording was too strong. Time-outs following incorrect responses are standard in Go/NoGo tasks as a way to discourage continuous licking rather than to deliver a formal "punishment." In our case, the time-outs were indeed longer than usual. Although we do not have formal behavioral proof that this specific duration was required, empirically we found that lengthening the time-out was the key change that reduced excessive licking and improved performance, whereas attempts to shorten it consistently increased false alarms. The relatively long baseline inter-trial interval (7 s), chosen to allow the hemodynamic response to return to baseline, likely contributed to this need for long time-outs: for a time-out to act as an effective deterrent in this context, it probably needs to be substantially longer than the standard inter-trial interval. Because this issue is peripheral to the main focus of the paper, we prefer not to develop it further in the Results or Discussion. We have, however, replaced the term "punished" with "discouraged" in the text to avoid implying a stronger conclusion than our data support.

11. In line 75, clarify if the stated n-values refer to total Difficult-Ref or Easy-Ref trials, or specifically to False Alarms within those categories.

Thanks. We have clarified this by adding the word “Target” or “Reference” as appropriate.

12. The title appears somewhat confusing, as the abstract explains that PMC encodes both real and reported auditory categories, depending on trial difficulty.

We appreciate this comment and agree that our wording in the abstract could be clearer. Conceptually, our claim is that PMC primarily encodes the internal (perceived) auditory category, not the overt report per se. On easy FA trials, our behavioral data suggest that the animal’s internal category remains “reference” while it nevertheless licks (a non-perceptual error). In this case, PMC activity follows the *perceived* (“reference”) category and diverges from the reported (lick/“target”) category. On difficult FA trials, perceptual errors are more likely; here the internal category is more often “target,” so PMC activity aligns with both the perceived and the reported category. In both cases, the most parsimonious interpretation is that PMC tracks the internal categorical belief, which sometimes, but not always, coincides with the report.
To avoid any ambiguity, we have revised the abstract to emphasize that PMC reflects the internal auditory category, which may or may not align with the reported choice depending on trial difficulty, bringing the text in line with the title.

13. Remove the comma in the abstract's final phrase: "motivational information."

Thanks. Done.

14. Clearly define "False Alarms" and "AM" upon first mention.

Thanks for catching this. We have done this.

15. Differentiate colors for Difficult-Ref and Easy-Ref FAs from the persistent vs. restrained licking categories in Supplementary Figure 4C-F. There is currently inconsistent color coding and figure layouts across these figures.

Thank you for pointing this out. After revisiting Supplementary Fig. 4C–F, we realized that these panels were largely redundant with Fig. 3A and did not add substantial new information beyond what is already shown more clearly there. To streamline the presentation and avoid potential confusion from inconsistent color coding, we have removed this supplementary figure. In parallel, we have added a new supplementary figure that more directly addresses licking-related analyses and other comments (in particular comment #7).

Reviewer #3 [Grace Ross]: High level summary: In this manuscript, the authors investigate whether neural activity in the ferret PMC - measured by hemodynamic response - reflects stimulus categorization or action in perceptual decision making during a head-fixed perceptual decision auditory task. The authors present compelling evidence that the hemodynamic responses in PMC differ based on perceptual difficulty and trial outcome, however additional analysis is needed to fully understand how PMC hemodynamic response differs in Easy and Difficult trials and an expanded discussion on how perceptual vs non-perceptual errors are classified in this task design is necessary to clarify the paper's conclusions. The argument that Easy task is more prone to non-perceptual errors is very interesting, and it make sense that the reason for an error in an Easy vs Difficult task is different, however the authors don't provide thorough behavioral evidence or interpretation to be convincing what the source of non-perceptual errors are.

Thank you for your review. We have added a dedicated cartoon as a new Figure 1A (in response to one of your later comments) that clarifies how perceptual and non-perceptual errors are classified. Regarding the differing sources of error in easy and difficult trials, we lean on prior literature, as laid out in the introduction through citations 1-10. This we believe is sufficient to demonstrate that perceptual errors should be more likely in difficult trials. We do not distinguish between the different types of nonperceptual error in this study, but it is not necessary to do so to reach our conclusions.
We respond to your point about how PMC hemodynamic responses differ when you mention it again below.

INTRODUCTION
Comment: I would consider a visual representation of the tested hypothesis with the experimental design, it gets quite confusing to think through the different conditions and what is predicted. A diagram would make this easier for the reader - maybe an addition to Figure 1?

Thanks for your feedback. We liked this idea a lot; this is now Figure 1A:

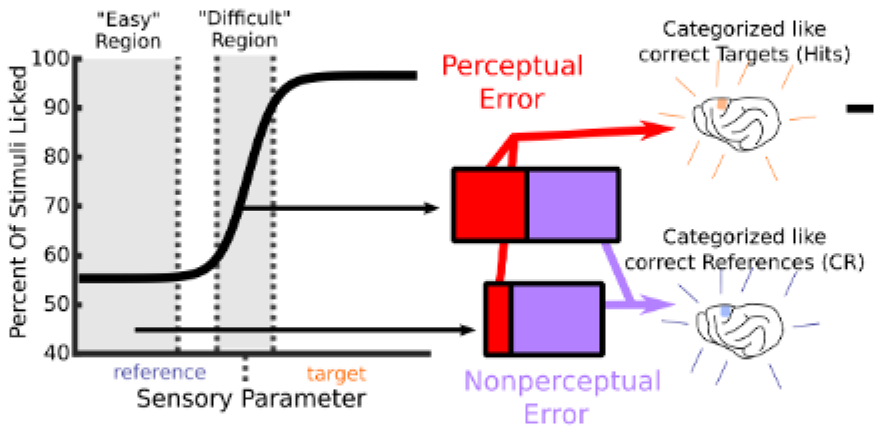

Comment: When writing the hypothesis earlier in the introduction, clearly refer to it as the 'stimulus category hypothesis' or reword in line 56, with the name in quotes I feel like it's referring back to something that was defined earlier in the text and interrupts flow

Thanks. Good point. We have changed this as follows:

> *False Alarm (FA) errors (i.e., incorrect "Go" responses to Reference stimuli) were also similar to CR trials, but only in Easy trials, in line with the "stimulus category" hypothesis presented above.*

Comment: Lines 54 to 58 I would be precise when making comparisons here, when the text says something like 'FA errors in Easy trials were also similar to CR trials…." it's not clear what similarity you're referring to, if you're talking about number of FA and CR, or the hemodynamic response. It does become clear in the next couple of sentences that you're discussing the hemodynamic response, but upon first impression it is unclear. Please edit language to be more precise.

Thanks. Another good point, we have edited the relevant section to make this clearer:

> *Hemodynamic response patterns for Easy and Difficult CR were similar, confirming that, when correct, Easy and Difficult trials showed categorical responses in PMC. Meanwhile, False Alarm (FA) errors (i.e., incorrect "Go" responses to Reference stimuli) were also similar to CR trials, but only in Easy trials, in line with the "stimulus category" hypothesis presented above.*

Comment: Introduction What is known about the primate/rodent/human prefrontal response to perceptual versus non-perceptual errors? I would contextualize open questions in the ferret with other model organisms and humans to further demonstrate/detail the need for this study. I also think that when working with a less common model organism like the ferret, context is important and can add a lot of relevance to your manuscript.

Thank you for this very helpful suggestion. To our knowledge, there is no prior work that explicitly operationalizes perceptual vs non-perceptual errors in the precise way we do here (i.e., linking on stimulus difficulty and action biases with neurophysiological measurements) in any species, including primates, rodents, or humans. We have not been able to identify studies that directly dissociate these two error types using a comparable behavioral definition. We have added a sentence to this effect, which should also help people contextualize the work as you request:

> *Such logic could be applied to this question in any animal model and brain region with categorical activity, and to our knowledge has not yet been applied in any other instance.*

RESULTS

Comment: Line 72 Please list stats for difficult stimuli as with easy stimuli

Thanks for your comment. We have modified the text accordingly:

> *Meanwhile, ferrets performed worse but decently on the difficult stimuli, as intended (ferret A: d' = 0.51, Difficult FA rate 0.79 ± 0.03, n = 193 trials, Difficult Hit rate 0.91 ± 0.02, n = 183 ; ferret B: d' = 1.1, Difficult FA rate 0.59 ± 0.03, n = 235 trials, Difficult Hit Rate 0.90 ± 0.02, n = 228; Figure \ref{fig1}C).*

Comment: are there enough non correct outcomes in the easy trials to be powered? Please provide a comprehensive breakdown of performance percentage's of each trial outcome for each condition, for both animals.

Thanks for your comment. Yes, there are ample non-correct outcomes in the easy conditions; this is in fact a major advantage of using an appetitive Go/NoGo paradigm with a motorized spout and delay, which together produce substantial false alarm rates. In practice, the limiting factor for power is the number of difficult trials, which we present less frequently, not the number of easy FAs. We did provide the counts of each kind of trial in lines 75-76 of the original text, however, as mentioned by another reviewer, we are not clear enough that these “n”s are the false alarm trials, as opposed to the total number of easy/difficult trials. We have clarified this in the text.

Comment: Figure 1E - confused how accuracy can be predicted along the entire time axis? I think this method needs more explanation of what the graph is actually showing… I'm still not sure what 'latency' is being referred to in line 94 and how what is in the graph relates to the hemodynamic response?

Thank you for this comment. We believe you are referring to Fig. 1I,J rather than Fig. 1E, as those panels show classification accuracy over time.
In these plots, we train a simple linear classifier independently at each time point using the CBV values from all PMC columns at that time. We then plot, as a function of time, the cross-validated decoding accuracy of that time-specific classifier. Thus, the curve does not mean that a single model predicts accuracy along the entire time axis; rather, it shows how well stimulus/outcome can be decoded from PMC activity at each time point in the trial.
The ‘latency’ refers to the time of these decoding peaks relative to stimulus onset. Because CBV responses lag underlying neural activity, one should interpret a decoding peak at time t as reflecting neural events roughly ~2 s earlier. We now make this explicit in the Results and clarify the link between classifier time-courses and the hemodynamic delay:

> *Models were trained on each time bin and slice separately, allowing for tracking of decoding accuracy over the trial timecourse. Hit versus CR decoding accuracy became significant 2 seconds after stimulus onset and plateaued approximately 1 second later. This latency aligns well with the expected timescale of fUSI hemodynamics; the*

hemodynamic response function (HRF) for fUSI has previously been reported as peaking ~2s post-neural activity onset (Nunez-Elizalde et al .2022, Bimbard et al 2018).

Comment: Figure 1E: is there a significance test that can be done at time -1 between Hit and CR? Or I would at least acknowledge in the text that even before the stimuli, there is a visible difference between Hit and CR, why that is and what that could mean

Thanks for your response. Another reviewer also mentioned this; we think it is a result of our choice in colormap, which allows small fluctuations to be perceptible. Quantitative proof that this difference is insignificant is already present in the paper: we trained the Easy Hit vs CR Classifiers at all time points, including baseline, and the classifiers were not above chance for pre-baseline periods in any consistent manner. This can be verified for all ferrets and slices using the left column of Supp Figures 3 and 4.

Comment: Section 3.3 How has the relationship between perceptual and non-perceptual errors and the Easy and Difficult task conditions been shown? Not controlling for a marker of attention or motivation, am I missing something or is this an assumption that's being made? I think explicit evidence is needed for this statement to hold more weight, especially as it is a primary part of this manuscript

Thank you for raising this important point. Our interpretation is indeed probabilistic, not categorical: we do not claim that all Easy-Ref FAs are non-perceptual and all Difficult-Ref FAs are perceptual. Rather, we rely on standard psychophysical reasoning (outlined in the Introduction) that errors on easy, saturated parts of the psychometric function are predominantly lapse-like/non-perceptual, whereas errors increase on the sloped part because perceptual uncertainty adds an additional source of mistakes. In other words, Difficult-Ref FAs are expected to contain the same pool of non-perceptual errors plus an extra contribution of perceptual errors, not to be purely perceptual.
We fully agree that we do not control a direct trial-by-trial marker of attention or motivation, and we now state this explicitly as a limitation in Section 3.3. Fluctuations in attention/motivation will contribute to the non-perceptual error pool across both Easy and Difficult conditions. What our data support is a relative enrichment of perceptual errors among Difficult-Ref FAs, consistent with the change in FA rates across difficulty and with the position of these stimuli on the psychometric slope.

Changes to section 3.3 include:

As laid out in the introduction, False Alarms in the steep region […]

To redirect the reader to the justification. The limitation is described at the end of the section as follows:

Note that, across difficulty levels, the total error rate reflects a mixture of perceptual and non-perceptual errors, with the proportion of perceptual errors increasing with difficulty

but never reaching 100%. Even so, this shift in the underlying mixture is detectable in the mean PMC response, leading to different average activity patterns for Easy versus Difficult False Alarms (Figure 1A).

Comment Fig2 B: If easy FA and easy CR look similar, but easy hit looks quite different, then the argument is that the animal can detect the reference stimuli but chooses sometimes to respond and other times not to. But when the target stimuli is present, this response is much higher because it's reward anticipatory? What is the argument that the animal is gaining by responding to the reference stimuli even though it's properly classified?

Thank you for this thoughtful comment. Your interpretation of Fig. 2B is close to our intended one: for Easy trials, the similarity between Easy-Ref FAs and CRs suggests that the internal category is “reference” in both cases, and that PMC activity differentiates from Hits (internally “target-like” and reward-predictive) for both types of reference trials. The larger PMC response on Easy Hits can reflect categorical coding (“target”), reward anticipation or both.
Regarding what the animal “gains” by responding to a correctly classified reference, we see two plausible contributions, both of which we had developed in the introduction but did not refer back to. First, from a normative perspective, occasional responding to reference stimuli can be viewed as exploration: if task contingencies or stimulus–reward mappings might change, the only way to discover this is to occasionally test the “wrong” action. In this sense, some Easy-Ref FAs may be deliberate exploratory licks.
Second, and likely more prominent in our Go/NoGo head-fixed appetitive setting, is a maladaptive action bias toward the rewarded “Go” response: animals may correctly classify the stimulus as reference but still fail to restrain licking due to a strong bias for the action that sometimes yields reward. This possibility is introduced in the Introduction (lines 18–30) and supported by prior work on lapse-like and action-biased errors in decision tasks (citations 4–8, 10).
In response to one of your later comments, we have additionally added text on how each of these kinds of non-perceptual error might relate to the PMC neurally. We hope this, in addition to the added reference back to the introduction in section 3.3, should provide sufficient context to the reader.

Comment Fig2C: the animal's PMC response is similar for a correct target trial and a difficult FA so the argument is that the animal thinks the tone they're responding to is correct because it matches to the Easy hit. Why isn't the Difficult FA and Difficult Hit plotted together? I would like to see that, as I think it's more informative because the comparison between an easy hit and difficult FA is not direct because the experimental parameters are different. I guess this is in E, but the metric is different so it doesn't feel like a clear comparison? I would still plot it and see if it's informative

Thank you for this suggestion. Our original choice in Fig. 2C was to keep the plot as simple as possible by showing Easy Hits (the canonical “target” response) together with Easy and Difficult FAs and CRs, and to quantify these conditions together in Fig. 2E. We did not include Difficult Hits because, in our data, their PMC time courses are essentially indistinguishable from Easy

Hits, and adding them made the main panel visually cluttered without changing the interpretation.
Following your comment, we have now added a supplementary panel in which Difficult Hits and Difficult FAs are plotted together (alongside CRs) using the same projection metric (in Supp Figures 3 and 4). These plots show that Difficult Hits overlap closely with Easy Hits, while Difficult FAs are shifted toward the Hit-like trajectory and away from CRs, consistent with our interpretation that many Difficult FAs reflect trials in which the internal category is “target-like.” We also plot the Difficult Hits mean responses in the new Supp Figure 6. We therefore keep the main text of Figure 2C focused on the cleanest comparison but now include the new supplementary figures for the direct Difficult Hit vs Difficult FA visualization you requested.

Comment Fig2D: A difficult CR where the animal identifies the reference stimuli and abstains is the same as in the Easy task. So the argument is that regardless of task condition, the response from PMC when inhibiting a response to the non-target stimuli is the same. Does this make sense, in terms of the role of prefrontal regions in responding differentially in task difficulty comparisons? Maybe that's dlPFC, but in PMC, I would discuss what this means because I'm surprised by this result - but maybe I'm misunderstanding

Your interpretation is essentially correct, and we agree this is an interesting aspect of the data. In our framework, this pattern makes sense if PMC activity primarily reflects task-dependent categorical state rather than difficulty per se following previous literature (Fritz et al, 2010, Yin et al, 2020). A Difficult CR and an Easy CR share the same internal category (“reference / no-go”). Once the animal has produced a CR, especially on a difficult trial, it is unlikely that the stimulus was misperceived, so the internal categorization is presumably correct in both cases.
In that view, it is not surprising that PMC responses for Easy and Difficult CRs largely overlap: PMC is signaling “this is a reference, do not act,” rather than encoding how hard it was to reach that decision. Difficulty- or confidence-related signals may be more strongly expressed in other prefrontal or associative areas (e.g. dlPFC or parietal cortex; see Zhou et al, 2016. *Scientific reports*), which we do not image here.

Comment Fig2E: from 3.4 to 5.4 seconds post stimulus onset. So a difficult FA elicits the strongest PMC response compared to a difficult CR and an Easy FA. I want a comparison of Difficult FA, Difficult CR and Difficult Hit. And if a false alarm is eliciting the largest reaction in PMC hemodynamics - if I'm understanding this graph correctly - then that should be thoroughly discussed. I'm not sure I agree with the choice of these comparisons. Fig2B needs to be replicated for Difficult trials. And looks like the ramp up of activity is only in trial outcomes that elicit a response, so more of an anticipatory signal than anything else?

Thank you for this detailed comment. Difficult Hits in our data are essentially superimposed on Easy Hits for both animals; they do not introduce a new pattern but add an almost identical trace. For clarity, we therefore kept Fig. 2B–D focused on Easy Hits as a canonical “target” trajectory and used Fig. 2E to summarize all conditions. Following your suggestion, we have now adjusted Supplementary Figures 5 and 6 to show Difficult Hits, Difficult FAs, and Difficult CRs together using the same projection metric. These confirm that Difficult Hits overlap closely

with Easy Hits, while Difficult FAs are shifted toward the Hit-like trajectory and away from CRs, consistent with our interpretation that many Difficult FAs reflect an internal “target-like” category.
The main purpose of Fig. 2B–D is to compare each critical condition (Easy FA, Difficult FA, Difficult CR) to the Easy Hit vs Easy CR axis, which serves as our reference for “target-like” vs “reference-like” PMC representations. We found that splitting them in such a manner greatly increased the clarity, and so wish to avoid packing more into the figures in the main text. We hope directing readers to the supplementary plots in this case will be sufficient.
Regarding the “ramp up” and anticipation: note that Easy FAs, which also involve a lick response, do not show a strong ramp in PMC (Fig. 2B), and this remains true when we control for licking intensity and pattern (Figs. 3D, 4D,F). The strong late PMC response appears on trials where the internal category is “target-like” (Hits and, probabilistically, Difficult FAs), not on all trials with a movement. Thus, the most parsimonious interpretation is that the ramp reflects a target-related state that is closely tied to reward-predictive categorization, which we discuss as PMC encoding internal category and its associated reward expectation, rather than a generic motor or anticipatory signal present whenever the animal licks. We now discuss this topic more in depth in the discussion section, interleaving the topic within a section we wrote in response to your comment 4.1. The relevant text is as follows:

> *However, fUSI measures neural activity indirectly through blood flow changes rather than directly from neuronal firing. Thus, our findings reflect integrated neural population responses rather than individual neuron activity. In line with previous work showing that neuronal response properties in ferret frontal cortex are varied and not strictly anatomically localized (Fritz et al 2010), it is likely that the region of PMC we studied contains mixed-selectivity neurons jointly sensitive to sensory category, action and reward expectation, as previously reported in rodents and primates (Reinert et al 2021, Fan et al 2017). Our data demonstrate that at the hemodynamic level PMC activity tracks the animal’s internal auditory category, but they cannot specify the relative contribution of these different neuronal subpopulations.*
> *A natural next step is therefore to use targeted electrophysiology guided by these fUSI maps. Based on the present results and existing work in frontal cortex, we would expect single-unit and population recordings in ferret PMC to reveal neurons whose firing (i) tracks the internal auditory category even when report and category diverge (e.g. Easy FAs), and (ii) more closely reflects reward anticipation or motor preparation. Our fUSI data identify specific PMC columns and time windows where such signals are likely to be strongest. Additionally, such a study could make use of a two-alternative forced choice paradigm, in which both sensory categories are rewarded to better dissociate sensory category signals from pure reward expectation, albeit potentially at the cost of reducing the number of non-perceptual errors that our current Go/NoGo design exploits.*

Comment: 3.4 Is an anticipatory response considered perceptual or non-perceptual?

Thank you for this question. As discussed above, “perceptual vs non-perceptual” refers in our framework to the origin of the error (misclassification of the stimulus vs lapse/action bias), not to whether a signal is anticipatory or not.

If by “anticipatory” you mean anticipation of reward, then such a signal would arise because the animal has internally categorized the stimulus as “target-like” and thus predicts potential reward. In that sense it is downstream of a perceptual categorization and tightly coupled to it: we would expect it to appear on Hits and on the subset of Difficult FAs where the internal category is “target,” but not on Easy FAs where the internal category remains “reference.” This matches our data: Easy FAs and Easy CRs show very similar PMC trajectories, whereas Easy Hits show a distinct, larger response.
If instead “anticipatory” refers to anticipation of making a movement (lick), that interpretation is harder to reconcile with the data, because Easy FAs involve licking but do not show the strong ramp seen on Hits and Difficult FAs. Given this, the most parsimonious view is that PMC activity primarily tracks the internal category / reward-predictive state, with perceptual and reward-expectation components that we cannot fully separate here. Crucially, in neither case does the late PMC activity simply reflect the final overt decision: Easy FAs and Easy Hits differ strongly, while Easy FAs and Easy CRs are very similar.

DISCUSSION
Comment: 4.1 I would strengthen the translational relevance of what the fUS results add to our knowledge as a field and how the addition of ephys would further this understanding. Instead of simply stating that ephys investigations are needed, I would hypothesize what those results may look like based on what is known about the electrophysiological activity of the ferret frontal cortex as a whole and specifically PFC vs PMC. This section reads too similar to a limitation section without a broader discussion of (1) what specifically does fUS add to current understanding/why it was chosen over ephys, (2) what do you predict based on the literature to see from an ephys investigation and how those results would meaningfully contribute to current knowledge.

Thank you for this very helpful suggestion. We agree that this part of the Discussion should more clearly articulate what fUSI adds and how future electrophysiology could build on these results. First, we now explain more explicitly why fUSI was chosen and what it adds:

> *fUSI allowed us to obtain mesoscale, columnar maps of task-related hemodynamics across multiple anterior-posterior positions in and around PMC, with high spatial resolution and stable anatomical registration across many days compared to other hemodynamic-based techniques. This wide coverage is difficult to achieve with standard chronic electrode arrays in ferret frontal cortex, where simultaneously sampling multiple neighboring columns and re-targeting exactly the same sites across weeks, remains challenging. This allowed us to reliably track the same regions across days anatomically, allowing the aggregation of rarer error trials which made this study feasible.*

Second, we now hypothesize what electrophysiology might reveal. Based on the current fUSI results and existing work in frontal cortex, we would expect single-unit and population recordings in ferret premotor regions to reveal mixed-selectivity neurons that combine sensory category, action, and reward expectation. We therefore have expanded the Discussion accordingly, emphasizing the specific advantages and interpretative value of fUSI in this study,

and concrete, literature-based predictions for future electrophysiological work in ferret PMC, and how such studies would refine and deepen the mechanistic picture suggested by our current hemodynamic findings (text is the same as for Comment Fig2E, except the final sentence was omitted):

> *However, fUSI measures neural activity indirectly through blood flow changes rather than directly from neuronal firing. Thus, our findings reflect integrated neural population responses rather than individual neuron activity. In line with previous work showing that neuronal response properties in ferret frontal cortex are varied and not strictly anatomically localized (Fritz et al 2010), it is likely that the region of PMC we studied contains mixed-selectivity neurons jointly sensitive to sensory category, action and reward expectation, as previously reported in rodents and primates (Reinert et al 2021, Fan et al 2017). Our data demonstrate that at the hemodynamic level PMC activity tracks the animal's internal auditory category, but they cannot specify the relative contribution of these different neuronal subpopulations.*
> *A natural next step is therefore to use targeted electrophysiology guided by these fUSI maps. Based on the present results and existing work in frontal cortex, we would expect single-unit and population recordings in ferret PMC to reveal neurons whose firing (i) tracks the internal auditory category even when report and category diverge (e.g. Easy FAs), and (ii) more closely reflects reward anticipation or motor preparation. Our fUSI data identify specific PMC columns and time windows where such signals are likely to be strongest.*

Comment: 4.2 I think discussing other internal states such as attention and reward anticipation are important to this discussion section. There was no discussion of impulsivity prior and it feels a bit out of the blue. How does this relate to perceptual vs non-perceptual decision making? This may be a good place to discuss how you might begin to disentangle the sources of non-perceptual errors, which I think is missing

Thank you for this helpful suggestion. We have substantially revised the Discussion to address these points.
First, we removed the isolated use of the term impulsivity and now use a consistent framework of internal states (attention, arousal, reward anticipation, and bias toward preferred actions) as modulators of whether the PMC internal category signal is expressed as a lick or as withholding.
Second, in the section "Nature and neural origin of non-perceptual decision making" we explicitly link these internal states to non-perceptual errors. We relate our results to work showing slow, brain-wide action-bias signals that modulate decisions without altering sensory encoding, and propose that non-perceptual errors in our task arise when downstream circuits read out a relatively stable PMC category representation through these fluctuating states.
Finally, we now outline concrete ways to disentangle different sources of non-perceptual errors (choice bias, exploration, attentional lapses), by exploiting differences in licking patterns (restrained vs persistent licks) and suggesting future experiments that combine licking kinematics, independent state markers (e.g., pupil, heart rate), and targeted recordings in orbitofrontal and parietal areas. It reads as follows:

In contrast, persistent licks may be more sensitive to the animal's broader motivational or exploratory state (e.g. frustration, thirst), or to history-dependent exploratory strategies in parietal cortex. Our current data cannot cleanly decompose these contributions, but they suggest that PMC provides a relatively stable internal category signal, while non-perceptual errors reflect how this signal is gated by state-dependent circuits in other frontal and parietal areas. Future work combining licking kinematics with independent markers of internal state (e.g. pupil size, heart rate) and targeted recordings in orbitofrontal and parietal cortices will be needed to disentangle action bias, exploration and attentional lapses as distinct sources of non-perceptual errors.

Comment 4.3 Propose downstream circuits override PMC categorical signals, causing non-perceptual errors. I would orient the reader a bit more to this discussion section with an example following the first sentence. Why would restrained licks be reflexive rather than deliberate? I would have thought the opposite.

Thank you for this helpful comment. We realized that the paragraph did not clearly articulate the role of attention and reward anticipation. We have now rewritten this section to (i) place our findings in the context of recent frontal cortex work showing that auditory "target" stimuli are represented as generic change-of-behavior cues across appetitive and aversive tasks, and (ii) explicitly discuss how internal states such as attention, arousal, reward anticipation, and action biases could modulate the readout of PMC category signals. The revised Discussion now emphasizes that PMC appears to encode the internal auditory category and the need to change behavior, whereas non-perceptual errors likely arise from state-dependent downstream circuits that transform this representation into action.

These changes are quite extensive and encompass most of the final two sections:

PMC internal category signals and their functional roles

Our results add to a growing body of work showing that frontal cortex does not simply encode specific motor acts, but more abstract "change of behavior" signals. Recent electrophysiology in ferret frontal cortex (Yin et al 2025) found that single neurons respond more strongly and persistently to sounds that instruct a change of ongoing behavior (Target cues) than to sounds that signal maintaining the current action (Reference cues), even when the same stimuli cue opposite movements and carry opposite reward valence across tasks. In line with this, PMC hemodynamic responses track the internal auditory category that drives a change in behavior: CBV patterns for difficult FAs resemble Hits, whereas easy FAs resemble CRs, indicating that PMC activity reflects the category the animal believes is present, rather than the eventual overt response.
We note that, because fUSI relies on neurovascular coupling in much the same way as fMRI, these findings also have direct relevance for human cognitive neuroscience. Human imaging studies routinely decode choice, difficulty and confidence from prefrontal

and premotor BOLD signals (Rolls et al 2010 (NeuroImage), Gherman et al 2018), but typically treat all errors as a single category. Our data show that, at least in ferret PMC, hemodynamic activity primarily tracks the internal category (and associated change-of-behavior state) even when the overt report is wrong. This suggests that human studies could gain sensitivity and interpretability by explicitly manipulating and separating perceptual and non-perceptual errors (e.g. via difficulty and action-bias manipulations) instead of pooling them. Such approaches would be particularly appropriate in studies where higher levels of choice bias or exploratory choices are expected, as a lapse in attention may also involve a perceptual error depending on the lapse's timing.

Nature and neural origin of non-perceptual decision making

[...] combine a relatively stable representation of the inferred auditory category with fluctuating internal states, leading to inappropriate Go responses on some Reference trials despite correct the PMC categorization. This may particularly be the case for the single "Restrained Licks", which appear reflexive rather than deliberate. Such reflexive actions likely engage circuits closely connected to the medullary reticular formation, the licking pattern generator (as shown in cats but likely also in the closely related ferret) (Travers et al 1997). A potential candidate region which would engage this formation is the orbital frontal cortex (OFC). Being agranular frontal cortex closely connected to limbic structures, OFC could influence reward-driven response criteria. Notably, stimulation of frontal orbital regions in cats induces rhythmic licking (Kubo et al 1981). Unfortunately, these areas were outside our imaging window. Future research should directly investigate these frontal-orbital and parietal regions to clarify their comparative roles in decision-making across species. In contrast, persistent licks may be more sensitive to the animal's broader motivational or exploratory state (e.g. frustration, thirst), or to history-dependent exploratory strategies in parietal cortex (Akrami et al 2018, Hwang et al 2017, Hwang et al 2019). Our current data cannot cleanly decompose these contributions, but they suggest that PMC provides a relatively stable internal category signal, while non-perceptual errors reflect how this signal is gated by state-dependent circuits in other frontal and parietal areas. Future work combining licking kinematics with independent markers of internal state (e.g. pupil size, heart rate) and targeted recordings in orbitofrontal and parietal cortices will be needed to disentangle action bias, exploration and attentional lapses as distinct sources of non-perceptual errors.

Comment: I would add a final discussion section about the impact of this research on the ferret neuroscience community, the prefrontal cortex community and - what I think is most missing - the human cognitive neuroscience community. These results are very interesting, what I think is missing is additional contextualization and answering the 'why does this matter' question a lot of readers may have.

Thank you for this very helpful comment. We agree that the original Discussion did not

sufficiently answer the “why does this matter?” question for readers working in ferret neuroscience and human cognitive neuroscience.
In the revised manuscript, we have added dedicated paragraphs that explicitly address these points. There, we first situate our results within the growing ferret frontal cortex literature, emphasizing for instance that our fUSI data complement recent electrophysiological work showing how ferret frontal cortex code actions, and formulate specific predictions about the nature of encoding that would be found with single neuron recordings in this regions. We now also make the translational link to human cognitive neuroscience: because fUSI and fMRI rely on the same type of hemodynamic signal, our results provide a concrete mechanistic template for interpreting decision-related BOLD activity in human frontal cortex, and specifically motivate human studies that manipulate and distinguish perceptual versus non-perceptual errors instead of pooling them. These changes were made simultaneous to many of the above changes (Comment 4.1 starting from “A natural next step” and 4.3 starting from “*We note that, because fUSI relies on neurovascular coupling”)*, so we do not reprint these changes here and refer you to the above.

Summary of specific figure requests/suggestions:
- Visual representation of hypotheses to improve readability
- I would like to see simple bar graphs of each ferret's performance in Easy and Difficult sessions, with percent of each trial outcome
- Fig 2B plot replicated for Difficult trials

Thanks. The first and third requests were addressed above. Figure 1C shows the performance of each ferret in easy and difficult trials. Easy and Difficult trials were not separated into different sessions, they were interleaved in the same sessions.
